# Biological mechanisms of sodium nitroprusside in enhancing quality of Radix Paeoniae Rubra and Radix Paeoniae Alba

**DOI:** 10.3389/fpls.2025.1660058

**Published:** 2025-11-12

**Authors:** Kai Zhao, Yafei You, Daiqian Deng, Qiujun Du, Zixian Guo, Lei Liu, Xiangcai Meng

**Affiliations:** 1 College of Pharmacy, Heilongjiang University of Chinese Medicine, Harbin, China; 2 College of Basic Medical Sciences, Mudanjiang Medical University, Mudanjiang, China

**Keywords:** Paeoniae Radix Rubra, Paeoniae Radix Alba, nitric oxide, reactive oxygen species, secondary metabolites, quality of medicinal herbs

## Abstract

**Introduction:**

Both Paeoniae Radix Alba (RAP) and Paeoniae Radix Rubra (RRP) are important botanical drugs used in Asian countries. Although they are both derived from the roots of *Paeonia lactiflora* Pall., they exhibit distinct pharmacological properties due to differences in germplasm and processing methods. Due to overwhelming market demand, the cultivated varieties have become the primary source to compensate for insufficient wild resources, which have led to decreased medicinal quality. This study aimed to address this quality decline and put forward a hypothesis that exogenous nitric oxide (NO) induces the reactive oxygen species (ROS)-mediated enhancement of secondary metabolism in fresh roots of *P. lactiflora*, thereby improving medicinal quality.

**Methods:**

Fresh roots of *P. lactiflora* germplasm for Paeoniae Radix Rubra production (RRP-germplasm) and for Paeoniae Radix Alba production (RAP-germplasm) were treated with sodium nitroprusside (SNP) at concentrations of 0.0, 0.1, 0.5, or 2.5 mmol/L to induce ROS bursts.

**Results:**

In the fresh roots of RRP-germplasm treated with 0.5 mmol/L SNP, the secondary metabolites paeoniflorin, albiflorin, oxypaeoniflorin, gallic acid, catechin, and paeonol were elevated by 19.1%, 205.4%, 115.4%, 19.9%, 201.0%, and 585.2%, respectively, and in the fresh roots of RAP-germplasm treated with 2.5 mmol/L SNP, the major secondary metabolites paeoniflorin, albiflorin, oxypaeoniflorin, gallic acid, catechin, and benzoic acid showed increases of 25.4%, 70.4%, 95.1%, 6.7%, 86.5%, and 33.6%, respectively. Moreover, experiments involving combined treatment with SNP and ROS scavengers demonstrated that ROS act as the key mediator linking exogenous NO to the secondary metabolism of *P. lactiflora*: scavenging ROS significantly attenuated the SNP-induced accumulation of target secondary metabolites.

**Discussion:**

Combined with the above findings of SNP promoting secondary metabolite synthesis, this study confirms that exogenous NO can improve the quality of cultivated RAP and RRP via ROS-mediated secondary metabolism, and clarifies the NO-ROS-secondary metabolism regulatory axis, offering insights for other medicinal plants’ quality improvement.

## 1 Introduction

Paeoniae Radix Rubra is primarily produced in Inner Mongolia and Hebei provinces in Northeast China, while Paeoniae Radix Alba is mainly produced in Zhejiang and Anhui provinces in China (Ma et al., 2024a). As bulk medicinal materials commonly used in Asian countries, these two herbs share the same botanical origin and medicinal part, and the Chinese Pharmacopoeia editions consistently classify the dried root of *P. lactiflora* as “Paeoniae Radix Rubra” and the dried root that goes through parboiling and removing its outer skin as “Paeoniae Radix Alba” (Pharmacopoeia Commission of the People’s Republic of China, 2020). In fact, Paeoniae Radix Rubra is derived from wild germplasm of *P. lactiflora*, whereas Paeoniae Radix Alba originates from the cultivated variety *P. lactiflora* ‘Baishao’ (Yao et al., 2020). The bioactive compounds, such as paeoniflorin, albiflorin, catechin, and paeonol, vary greatly between them (Fan et al., 2014; Liu et al., 2015). Paeoniae Radix Rubra contains higher levels of paeoniflorin, catechin, and paeonol, and its effects are mainly achieved by inhibiting the release of inflammatory factors such as tumor necrosis factor-*α* and interleukin-6, as well as scavenging ROS and other oxidative stress products, thereby exhibiting notable microcirculation-improving, analgesic, anti-inflammatory, antibacterial, and antiviral effects, and it is commonly used for treating hemorheological abnormalities, inflammatory conditions, and infection-related diseases. Paeoniae Radix Alba is richer in albiflorin, and its effects are mainly achieved by regulating the activity of T lymphocytes and B lymphocytes to enhance immune function, while promoting the expression of anti-apoptotic proteins in hepatocytes and inhibiting hepatocyte necrosis, thereby excelling in hematopoietic promotion, immune enhancement, hepatoprotection, and antitumor effects, and it is more suitable for patients with chronic anemia, immunodeficiency, hepatic disorders, and cancer (Zhang et al., 2013). A large demand for both Paeoniae Radix Rubra and Paeoniae Radix Alba makes the cultivated ones the predominant commercial source. However, the cultivated materials exhibit inferior quality; How to improve the quality of cultivated products has become crucial for enhancing clinical efficacy.

Notably, the medicinal components of traditional Chinese herbs are typically secondary metabolites, and environmental stress serves as a fundamental trigger for their biosynthesis of secondary metabolites in plants. Moderate environmental stressors can effectively promote the biosynthesis of secondary metabolites (Alami et al., 2024). Therefore, strategic application of environmental stress may rapidly improve the quality of herbal medicinal materials.

ROS are inevitable products of metabolic processes in living organisms. At appropriate concentrations, ROS are indispensable for the formation of disulfide bonds (-S-S-) in proteins (Tu and Weissman, 2004; Malhotra and Kaufman, 2007), contributing to the formation of the unique structures of enzymes and other functional proteins and regulating plant growth and development (Buchanan and Luan, 2005; Akter et al., 2015). Under normal physiological conditions, ROS are maintained at a relatively stable level. However, when plants are exposed to environmental stresses such as drought, waterlogging, salinity, heat shock, chilling injury, or UV radiation, disturbed metabolic activity leads to excessive production of ROS in chloroplasts, mitochondria, peroxisomes, and other organelles. During photosynthesis in chloroplasts, photosystem I generates increased superoxide anion (O_2_^·-^) through the Mehler reaction by transferring more electrons to O_2_ (Asada, 2006; Khorobrykh et al., 2020). In mitochondria, electron leakage from complexes I and III of the electron transport chain during oxidative phosphorylation for Adenosine Triphosphate (ATP) production partially reduces O_2_ to O_2_^·-^ (Hansen et al., 2006). Additionally, the photorespiration process involves glycolate oxidase catalyzing the oxidation of glycolate to glyoxylate, accompanied by the production of ROS such as H_2_O_2_ (Miller et al., 2010). ROS include O_2_^·-^, H_2_O_2_, ·OH, ^1^O_2_, etc (Droge, 2002). It has been proven that ROS accumulation is an inevitable consequence of environmental stress, with a 10-fold increase in H_2_O_2_ and a 3-fold increase in O_2_^·-^ under adverse conditions (Shen et al., 2020). The excessive generation of ·OH and O_2_^·-^ with high activity can readily trigger cascading oxidative damage, subsequently alter adjacent molecular structures, compromise biomembrane stability, cause DNA damage, break peptide chains, and induce protein cross-linking, disrupt metabolic pathways, and program cell death (Grimm et al., 2012; Van Ruyskensvelde et al., 2018; Xie et al., 2019). The immobility of plants inevitably leads to elevated ROS levels when exposed to environmental stress. Given the potent protein-damaging effects of ROS, which often overwhelm antioxidant enzymes under severe stress, the fundamental reason why plants can survive is that they have evolved secondary metabolism and utilize secondary metabolites, usually the active components of herbal medicine, to scavenge excess ROS. Therefore, the outbreak of ROS caused by environmental stress is a basic factor that enhances secondary metabolism and improves the quality of medicinal materials.

NO, a reactive nitrogen species (RNS), can induce the production of ROS such as O_2_^·-^ through redox cycles and modulation of RNS-ROS interactions (Heinrich et al., 2013; Del Río L, 2015), trigger physiological responses in plants under stress, and enhance the quality of medicinal materials. In contrast to the negatively charged O_2_^·-^, NO is an electrically neutral small molecule with both lipophilic and hydrophilic properties, and can freely traverse cell membranes and distribute widely in the cellular environment. Additionally, NO contains unpaired electrons, allowing it to react with O_2_^·-^ to form peroxynitrite, a less toxic compound that mitigates stress-induced damage in plants. These properties underscore the critical role of NO in plant growth, development, and environmental adaptation (Delledonne et al., 1998a). It has been demonstrated that exogenous NO stress can increase the biosynthesis of secondary metabolites in plants (Song et al., 2023; Zhang et al., 2025). SNP, a commonly used exogenous NO donor, contains a labile Fe-NO bond in its molecule, enabling the rapid release of a large amount of NO.

Against this background, to investigate the biological mechanisms of SNP in enhancing the quality of Radix Paeoniae Rubra and Radix Paeoniae Alba, this study treated isolated fresh roots of *P. lactiflora* with SNP solutions of different concentrations, and its research contents include: (1) investigating the effect of exogenous NO on O_2_^·-^ and H_2_O_2_ to verify whether ROS are products of NO-induced stress; (2) examining the effect of exogenous NO on MDA to verify whether NO can induce the physiological effects of environmental stress; (3) exploring the effect of exogenous NO on antioxidant enzymes to investigate the patterns and the limitations of these enzymes in scavenging ROS; (4) assessing the effect of exogenous NO on secondary metabolism to verify whether NO can regulate metabolic pathways and enhance secondary metabolism; and (5) evaluating the effect of ROS scavengers on the reversal of NO-induced effects to confirm whether ROS act as the key mediators linking exogenous NO to the secondary metabolism of *P. lactiflora*. This study centers on medicinal material quality, using fresh medicinal parts to clarify the association between environmental stress and secondary metabolism, elucidate the formation mechanism of the quality of medicinal materials, and explore new approaches to improving quality.

## 2 Materials and instruments

### 2.1 Materials

The experimental materials were obtained from four-year-old cultivated RRP-germplasm and RAP-germplasm of *P. lactiflora*, grown at the medicinal herbs production base in the Greater Khingan Mountains region of Heilongjiang Province, China. The fresh roots were harvested on October 5, 2024, with more than 10 intact plants of each variety selected as research subjects, totaling 15 kg of fresh roots, authenticated by Professor Xiang-cai Meng, Heilongjiang University of Chinese Medicine.

### 2.2 Reagents

Protein quantification (TP) assay kit, H_2_O_2_ assay kit, MDA assay kit, superoxide dismutase (SOD) assay kit, catalase (CAT) assay kit, peroxidase (POD) assay kit, and phenylalanine ammonia-lyase (PAL) assay kit (Nanjing Jiancheng Bioengineering Institute, Nanjing, China, batch numbers: 20241231, 20250103, 20250106, 20241226, 20250104, 20241231, and 20241106, respectively); plant 1,3-bisphosphoglycerate (1,3-DPG) enzyme-linked immunosorbent assay (ELISA) kit and plant 3-Hydroxy-3-Methylglutaryl-CoA reductase (HMGR) ELISA kit (Jiangsu Jingmei Biotechnology Co., Ltd., Nanjing, China, batch numbers: 20250228 and 20250409, respectively); O_2_·^-^ level detection kit (Beijing Solarbio Science & Technology Co., Ltd., Beijing, China, batch number: 2501001001); methanol (analytical grade, Tianjin Fuyu Fine Chemical Co., Ltd., Tianjin, China, batch number: 20240509); phosphoric acid (analytical grade, Tianjin Hengxing Chemical Reagent Manufacturing Co., Ltd., Tianjin, China, batch number: 20240406); acetonitrile (HPLC grade, Beijing Dikma Technologies Inc., Beijing, China, batch number: 20240318); sodium nitroprusside (Zhengzhou Pini Chemical Reagent Factory, Zhengzhou, China, batch number: 20240521); glacial acetic acid (analytical grade, Tianjin Tianli Chemical Reagent Co., Ltd., Tianjin, China, batch number: 20231108); paeoniflorin, albiflorin, oxypaeoniflorin, gallic acid, catechin, paeonol, benzoic acid, and benzoylpaeoniflorin (Chengdu Alfa Biotechnology Co., Ltd., Chengdu, China, batch numbers: AFCE0452, MRDE0804, AFCC0904, AFDG1553, AFBF2708, AFBG1209, AFCJ1302, and AFCC0952, respectively; purity ≥98.0%); physiological saline (Harbin Sanlian Pharmaceutical Co., Ltd., Harbin, China, batch number: 20241102); phosphate buffer (pH 7.2, Shanghai Aladdin Biochemical Technology Co., Ltd., Shanghai, China, batch number: 20241219); *α*-tocopherol (Shanghai Aladdin Biochemical Technology Co., Ltd., Shanghai, China, batch number: 20250904); and N-acetyl-L-cysteine (Guangzhou Chemical Reagent Factory, Guangzhou, China, batch number: 20250820).

## 3 Methods

### 3.1 Sample handling

#### 3.1.1 Treatment of fresh roots of *P. lactiflora* with different SNP concentrations

Fresh, intact roots of RRP-germplasm and RAP-germplasm were divided into four groups according to diameter (within ±0.5 cm ranges), length (within ±5 cm ranges), and weight (within ±50 g ranges). An appropriate amount of fresh samples were selected, surface soil was cleaned off, and the samples were uniformly sprayed with SNP solutions at concentrations of 0.0 (CK), 0.1, 0.5, and 2.5 mmol/L, respectively. Spraying was performed every 8 hours until the surfaces of fresh roots reached water saturation, and the entire process was conducted in the dark for 3 days. The samples were collected on the 0^th^, 1^st^, 2^nd^, and 3^rd^ days, with a sampling interval of 24 h. The sampling methods were as follows: (1) Each sample was derived from at least 5 plants. Using an AG135 analytical balance (0.1 mg precision; METTLER, Switzerland), precisely 50 aliquots of 0.3 g each were weighed and stored at -80°C in a freezer. These aliquots were used for the determination of O_2_^·-^, H_2_O_2_, MDA, and 1,3-DPG contents, as well as the activities of SOD, CAT, POD, PAL, and HMGR. During the determination process, absorbance values were read using a Thermo microplate reader (Thermo Inc., USA) to calculate the content of each index. (2) Fresh roots (>200 g per group) were used: for RRP-germplasm, after removing root tips, root bases, and fine roots, the roots were sun-dried; for RAP-germplasm, after the same pretreatment, the roots were boiled in water, peeled, and then sun-dried. The dried roots were pulverized and passed through a No. 3 sieve, and these powdered samples were used for the determination of the contents of paeoniflorin, albiflorin, oxypaeoniflorin, gallic acid, catechin, paeonol, benzoic acid, and benzoylpaeoniflorin. Quantitative analysis was performed using a Model 1200 HPLC (Agilent Technologies Inc., USA). All samples were processed in triplicate.

#### 3.1.2 Combined treatment of fresh roots of RRP-germplasm with SNP and ROS scavengers

Additional fresh roots of RRP-germplasm meeting the same selection criteria as Section 2.1.1 were divided into four groups: water control group (CK), SNP treatment group, SNP +*α*-tocopherol (*α*-Toc) group, and SNP + N-acetylcysteine (NAC) group. The CK group was sprayed with distilled water; the SNP treatment group was sprayed with 0.5 mmol/L SNP solution; and the combined groups were first sprayed with 0.5 mmol/L SNP solution, followed by 0.1 mmol/L *α*-Toc or 1.0 mmol/L NAC solution (2-hour interval between sprays). For all groups, spraying was performed every 8 hours until the solution was about to drip, with the entire process conducted in the dark for 3 days. Samples were collected on Days 0, 1, 2, and 3 (24-hour intervals), following the sampling protocol in Section 2.1.1 with modifications as follows: (1) Each sample was derived from at least 5 plants. 30 aliquots of 0.3 g each were precisely weighed and stored at -80°C. These aliquots were used only for determining O_2_^·-^, H_2_O_2_, and MDA contents. (2) Fresh roots (>200 g per group) were processed by removing root tips, root bases, and fine roots, then sun-drying, pulverizing, and passing through a No. 3 sieve. These powdered samples were used for determining paeoniflorin and 7 other components. The instruments used in this experiment were the same as those in Section 2.1.1. All samples were processed in triplicate.

### 3.2 Determination of ROS level

The levels of O_2_·^-^ and H_2_O_2_ are determined using O_2_·^-^ detection kits and H_2_O_2_ assay kits (Zhang et al., 2025), with the results expressed in μmol/g and mmol/g, respectively.

### 3.3 Determination of MDA level

The MDA content in fresh roots is quantified using an MDA assay kit with a thiobarbituric acid (TBA) method (Zhang et al., 2025), and the results are reported in nmol/g.

### 3.4 Measurement of antioxidant enzyme activities

The activities of SOD, POD, and CAT in fresh roots are assessed using their respective assay kits (Zhang et al., 2025), with all results expressed in U/g.

### 3.5 Determination of 1,3-DPG level

The 1,3-DPG content in fresh roots is measured using a plant-specific 1,3-DPG ELISA kit (Song et al., 2023), and the results are presented in μmol/L.

### 3.6 Measurement of PAL and HMGR activities

The activities of PAL and HMGR in fresh roots are evaluated using their respective assay kits (Shen et al., 2020; Song et al., 2023), with the results reported in U/g and μg/L, respectively.

### 3.7 Determination of secondary metabolite level

#### 3.7.1 Preparation of reference standard solutions

An appropriate amount of paeoniflorin, albiflorin, oxypaeoniflorin, gallic acid, catechin, paeonol, benzoic acid, and benzoylpaeoniflorin was accurately weighed and placed in a volumetric flask. The mixture was dissolved in 50% methanol to prepare a mixed reference standard solution containing the eight components. The final concentrations of these eight components in the solution were 1.058, 1.047, 0.687, 0.560, 0.550, 0.488, 0.753, and 0.525 g/L, respectively.

#### 3.7.2 Preparation of the test solution

0.20 g of the medicinal powder was accurately weighed and transferred into a stoppered conical flask. 20.0 mL of 50% methanol solution was precisely added, the flask was stoppered tightly, and subsequently weighed. The mixture was allowed to soak for 4 hours, followed by ultrasonication (200 W power, 40 kHz frequency) carried out for 30 minutes. After cooling, the flask was reweighed, and the lost mass was replenished with 50% methanol. The solution was centrifuged at 4000 r/min for 5 minutes, the supernatant was collected, and filtered through a 0.22 μm microporous membrane to obtain the final test solution.

#### 3.7.3 Chromatographic conditions

The separation is performed on a Diamonsil C_18_ column (250 mm × 4.6 mm, 5 μm) using a mobile phase consisting of acetonitrile (A) and a pH 2.7 phosphoric acid aqueous solution (B) under gradient elution as follows: 0~20 min, 5% A to 15% A; 20~40 min, 15% A to 20% A; 40~50 min, 20% A; 50~80 min, 20% A to 40% A; 80~90 min, 40% A to 5% A; 90~100 min, 5% A. The column temperature is maintained at 25°C, with an injection volume of 10 μL and a flow rate of 1 mL/min. Detection is carried out at a wavelength of 230 nm (**Supplementary Figure 1**).

#### 3.7.4 Methodological investigation

##### 3.7.4.1 Linear relationship

The prepared mixed reference solution as described in Section 2.7.1 was serially diluted with 50% methanol to prepare a series of concentrations. Under the aforementioned chromatographic conditions, the solutions were injected, and the peak areas of paeoniflorin, albiflorin, oxypaeoniflorin, gallic acid, catechin, paeonol, benzoic acid, and benzoylpaeoniflorin were recorded. Regression analysis was performed using the mass concentration as the abscissa (*X*) and the peak area as the ordinate (*Y*). The regression equations, coefficients of determination (*R²*), and linear ranges for the eight components were calculated, demonstrating good linearity within the specified ranges (**Supplementary Table 1**).

##### 3.7.4.2 Precision

The same sample solution was analyzed under the aforementioned chromatographic conditions with six replicate injections to determine intra-day precision, followed by analyzing it over three consecutive days to assess inter-day precision. The relative standard deviations (RSD) of intra-day and inter-day precision for the eight components were 1.80%, 1.19%, 0.49%, 0.72%, 1.08%, 1.42%, 0.84%, and 1.53%; and 1.41%, 1.10%, 0.69%, 0.56%, 0.97%, 0.76%, 0.53%, and 0.79%, respectively, demonstrating excellent precision.

##### 3.7.4.3 Stability

The same sample solution was analyzed at 0, 2, 4, 8, 12, and 24 h under the aforementioned chromatographic conditions. The RSD values of the eight components were 1.24%, 0.94%, 0.81%, 0.82%, 1.32%, 1.08%, 0.81%, and 1.09%, respectively, demonstrating that the sample solution remained stable within 24 h.

##### 3.7.4.4 Reproducibility

Six sample solutions were prepared from the same test material and analyzed under the aforementioned chromatographic conditions. The RSD values for the eight components were determined to be 1.13%, 0.54%, 0.61%, 0.79%, 1.02%, 0.59%, 1.07%, and 0.95%, respectively, demonstrating excellent repeatability of the analytical method.

##### 3.7.4.5 Spike recovery test

Six portions of Paeoniae Radix Rubra powder were accurately weighed, and appropriate amounts of the corresponding reference standards of known concentration were added to each portion to prepare six test solutions. The samples were then analyzed under the established chromatographic conditions. The recovery rates of the eight components were determined to be 100.2%, 99.5%, 99.1%, 99.9%, 99.4%, 98.8%, 100.3%, and 98.2%, with corresponding RSD values of 1.08%, 1.38%, 1.22%, 1.56%, 0.91%, 0.85%, 1.26%, and 1.30%, respectively, demonstrating satisfactory recovery and accuracy with the proposed method.

#### 3.7.5 Data processing methods

The data were processed using Microsoft Office Excel 2021 (Microsoft Corporation, USA), and graphs were generated with Prism 8 (GraphPad Software, USA). All data are presented as mean ± standard deviation (Mean *±* S.D.). Statistical analyses were performed using independent samples *t*-tests in IBM SPSS 28.0 (IBM Corporation, USA). A statistically significant difference was defined as ^*^P< 0.05 or ^**^P< 0.01.

## 4 Results

### 4.1 ROS level in fresh roots of *P. lactiflora*

#### 4.1.1 ROS level in fresh roots of RRP-germplasm

**Figure 1:**
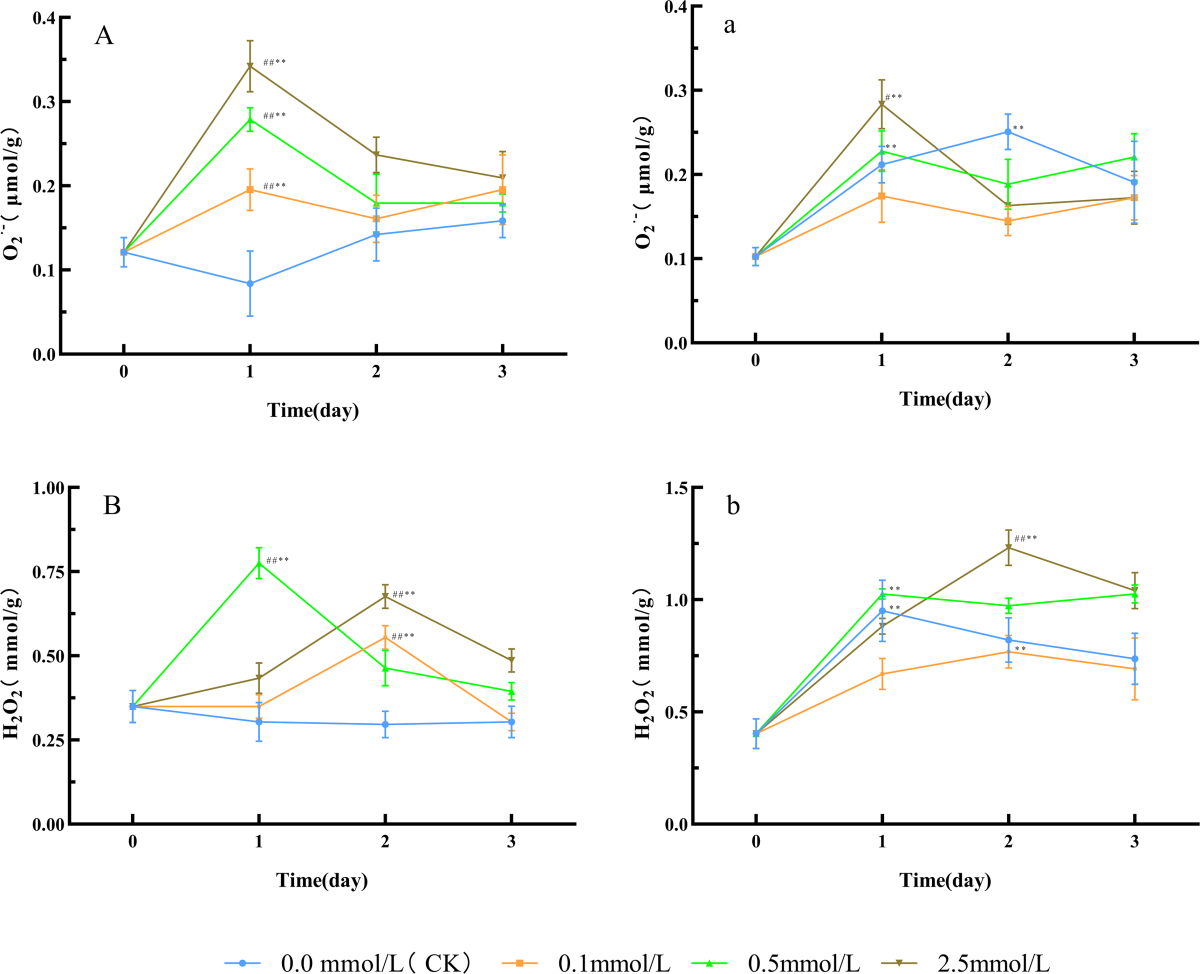
ROS level changes in Paeoniae Radix Rubra **(A, B)** and Paeoniae Radix Alba **(a, b)** under different SNP concentrations. Data are presented as $\;\bar{x}$*± s* (*n* = 3, error bars: SD). Significance: */** vs day 0 (*P < 0.05, **P < 0.01); #/## vs 0.0 mmol/L SNP (#*P *< 0.05, ## *P *< 0.01). Groups: 0.0 mmol/L SNP (CK, blue), 0.1 mmol/L SNP (orange), 0.5 mmol/L SNP (green), 2.5 mmol/L SNP (brown).A/a: O_2_·^-^ contents (μmol/g); B/b: H_2_O_2_ contents (mmol/g).

Compared with day 0, the O_2_·^-^ level in the water (CK) group showed no significant change trend, while all SNP-treated groups exhibited an initial increase followed by a decrease. The O_2_·^-^ level in the 0.1, 0.5, and 2.5 mmol/L SNP groups all peaked on day 1, showing an increase of 61.5%, 130.8%, and 182.7% respectively, with the 2.5 mmol/L SNP group showing the most pronounced elevation (**Figure 1A**). Similarly, compared with day 0, the H_2_O_2_ level in the water group showed no significant change trend, while all SNP-treated groups exhibited an initial increase followed by a decrease. The H_2_O_2_ level in the 0.1, 0.5, and 2.5 mmol/L SNP groups peaked on days 2, 1, and 2, respectively, showing increases of 58.7%, 121.7%, and 93.5%, with the 0.5 mmol/L group showing the most pronounced elevation (**Figure 1B**). The ROS levels in all SNP-treated groups were significantly higher than those in the CK group, indicating that SNP could induce ROS production.

#### 4.1.2 ROS level in fresh roots of RAP-germplasm

Compared with day 0, the 0.1 mmol/L SNP group showed no significant change in O_2_·^-^ level, while the other groups exhibited an initial increase followed by a decrease. The O_2_·^-^ level in the 0.0, 0.5, and 2.5 mmol/L SNP groups peaked on days 2, 1, and 1, respectively, with an increase of 145.5%, 122.7%, and 177.3%, respectively, and the 2.5 mmol/L SNP group showed the most pronounced elevation (**Figure 1a**). Regarding H_2_O_2_ levels, compared to day 0, all SNP-treated groups showed an initial increase followed by a decrease in H_2_O_2_ levels, with the most pronounced elevation observed in the 2.5 mmol/L SNP group. The H_2_O_2_ levels in the 0, 0.1, 0.5, and 2.5 mmol/L SNP groups peaked on days 1, 2, 1, and 2, respectively, exhibiting an increase of 135.8%, 90.6%, 154.7%, and 205.7% (**Figure 1b**). These results indicate that the ROS levels in all groups generally displayed an initial rise followed by a decline, and the increases were positively correlated with SNP concentration.

### 4.2 MDA level in fresh roots of *P. lactiflora*

#### 4.2.1 MDA level in fresh roots of RRP-germplasm

**Figure 2:**
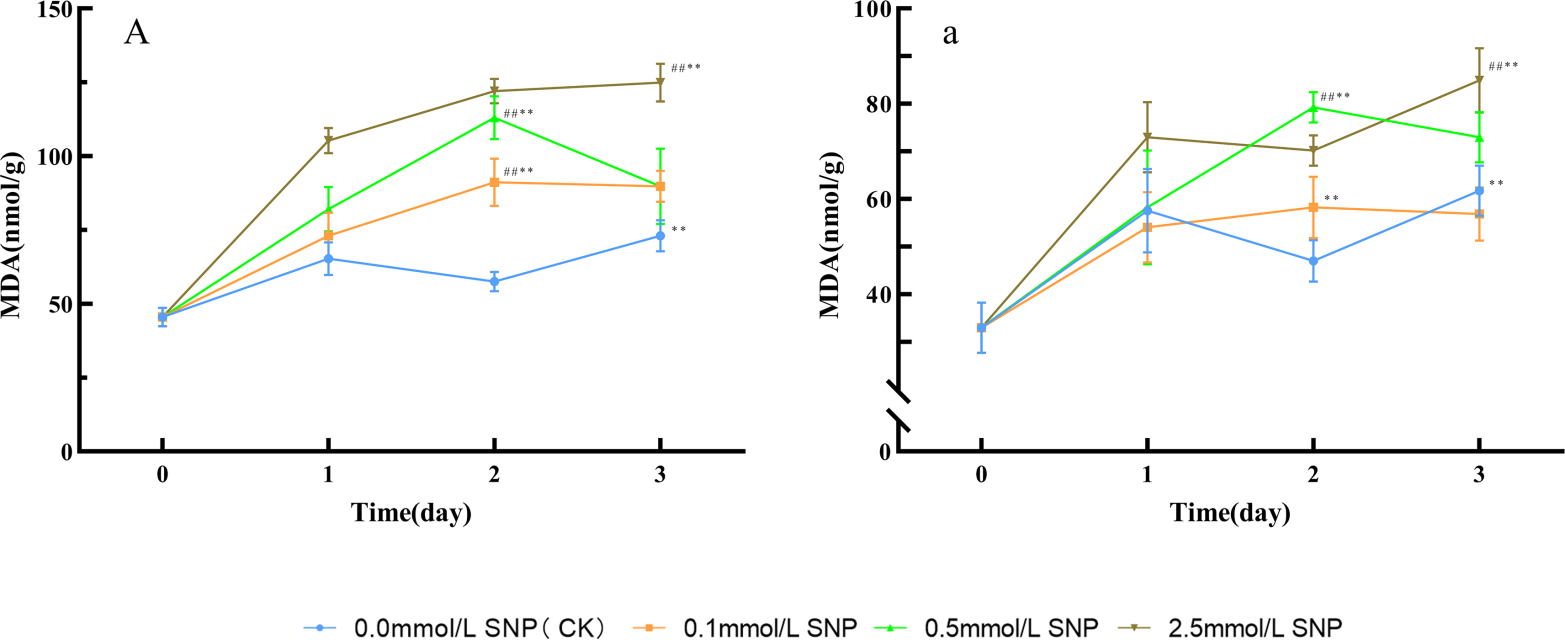
MDA content changes in Paeoniae Radix Rubra **(A)** and Paeoniae Radix Alba **(a)** under different SNP concentrations. Data are presented as $\;\bar{x}$*± s* (*n* = 3, error bars: SD). Significance: */** vs day 0 (*P < 0.05, **P < 0.01); #/## vs 0.0 mmol/L SNP (#*P* < 0.05, ##*P* < 0.01). Groups: 0.0 mmol/L SNP (CK, blue), 0.1 mmol/L SNP (orange), 0.5 mmol/L SNP (green), 2.5 mmol/L SNP (brown). A/a: MDA contents (nmol/g).

Compared with day 0, the MDA contents in the water (CK) group showed a slight increasing trend, while all SNP-treated groups exhibited greater increases than those in the CK group. The most pronounced elevation was observed in the 2.5 mmol/L SNP group. The MDA contents in the 0.1, 0.5, and 2.5 mmol/L SNP groups peaked on days 2, 2, and 3 of treatment, respectively, with increases of 100.1%, 147.8%, and 173.9% (**Figure 2A**). In all SNP-treated groups, the MDA levels were significantly higher than those in the CK group, indicating that SNP caused substantial cellular damage.

#### 4.2.2 MDA level in fresh roots of RAP-germplasm

Compared with day 0, the MDA contents in the water and 0.1 mmol/L SNP groups showed minor fluctuations, while the other two groups exhibited an upward trend, with the most significant increase observed in the 2.5 mmol/L SNP group. The MDA contents in the 0.5 and 2.5 mmol/L SNP groups peaked on days 2 and 3 of treatment, respectively, showing increases of 140.4% and 157.4% compared to day 0 (**Figure 2a**).

### 4.3 Antioxidant enzyme activities in fresh roots of *P. lactiflora*

#### 4.3.1 Antioxidant enzyme activities in fresh roots of RRP-germplasm

**Figure 3:**
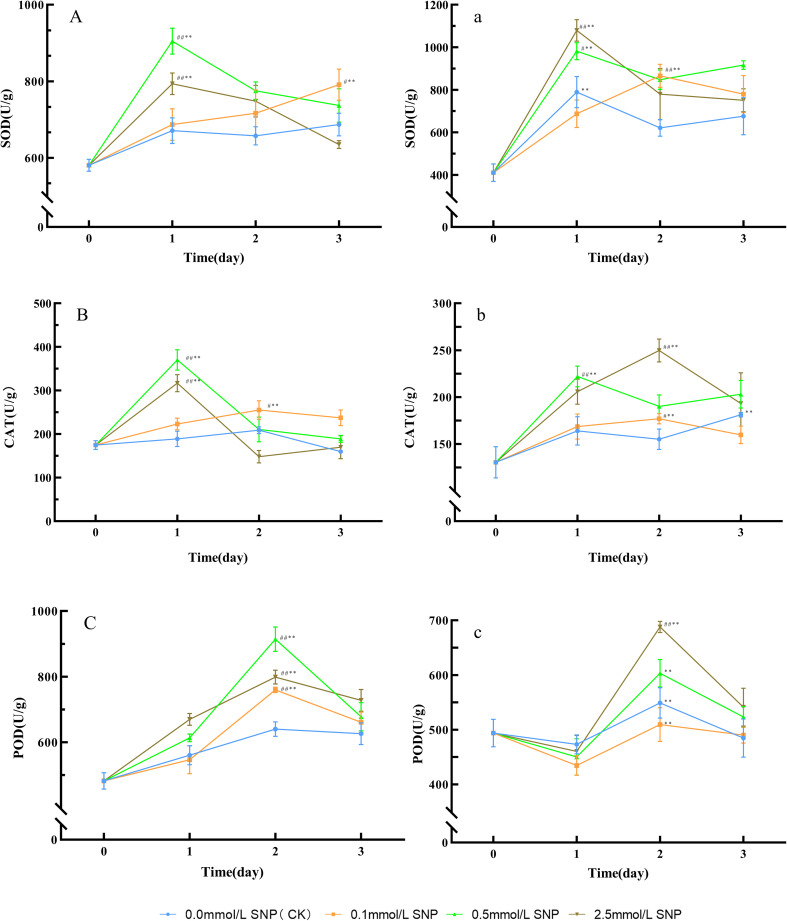
Antioxidant enzyme activities changes in Paeoniae Radix Rubra **(A–C)** and Paeoniae Radix Alba **(a–c)** under different SNP concentrations. Data are presented as $\;\bar{x}$*± s* (*n* = 3, error bars: SD). Significance: */** vs day 0 (*P < 0.05, **P < 0.01); #/## vs 0.0 mmol/L SNP (#*P* < 0.05, ##*P* < 0.01). Groups: 0.0 mmol/L SNP (CK, blue), 0.1 mmol/L SNP (orange), 0.5 mmol/L SNP (green), 2.5 mmol/L SNP (brown). **(A, a)**: SOD activities; **(B, b)** CAT activities; **(C, c)** POD activities (all units: U/g).

Compared with day 0, the SOD activities in the water (0 mmol/L SNP/CK) group showed no significant change trend. The 0.1 mmol/L SNP group exhibited a gradual increasing trend, while the other two groups had an initial increase followed by a decrease. The SOD activities in the 0.1, 0.5, and 2.5 mmol/L SNP groups reached their peaks on days 3, 1, and 1 of treatment, respectively, showing increases of 36.3%, 55.9%, and 36.7%, with the 0.5 mmol/L SNP group showing the most pronounced elevation (**Figure 3A**). Similarly, compared with day 0, the CAT activities in the water group showed no significant change trend, while all other SNP groups exhibited an initial increase followed by a decrease. Among them, the 0.5 mmol/L SNP group demonstrated the most pronounced elevation. The CAT activities in the 0.1, 0.5, and 2.5 mmol/L SNP groups peaked on days 2, 1, and 1, respectively, showing increases of 45.8%, 111.7%, and 81.1% (**Figure 3B**). Likewise, compared with day 0, the POD activities in the water group showed no significant change trend, while all other SNP-treated groups exhibited an initial increase followed by a decrease. The POD activities in the 0.1, 0.5, and 2.5 mmol/L SNP groups peaked on day 2, increasing by 57.6%, 89.6%, and 65.6%, respectively, with the 0.5 mmol/L SNP group showing the most pronounced rise (**Figure 3C**). It is not difficult to find that from days 1 to 3, the antioxidant enzyme activities in all SNP-treated groups were higher than those in the CK group, while the CK group showed almost no change, indicating that antioxidant enzymes play a crucial role in the early stages of SNP treatment.

#### 4.3.2 Antioxidant enzyme activities in fresh roots of RAP-germplasm

Compared with day 0, the SOD activities in all groups showed an initial increase followed by a decline. The SOD activities in the water, 0.1, 0.5, and 2.5 mmol/L SNP groups reached peaks on days 1, 2, 1, and 1 of treatment, respectively, with increases of 92.3%, 111.0%, 139.2%, and 163.0%, with the most pronounced increase observed in the 2.5 mmol/L SNP group (**Figure 3a**). Regarding CAT activities, compared with day 0, the CAT activities in the water and 0.1 mmol/L SNP groups showed minor fluctuations, while the other two SNP groups exhibited an initial increase followed by a decline. The CAT activities in the 0.5 and 2.5 mmol/L SNP groups peaked on days 1 and 2 of treatment, respectively, showing increases of 70.3% and 91.6%, with the 2.5 mmol/L SNP group showing the most pronounced elevation (**Figure 3b**). As for POD activities, compared with day 0, the POD activities in all treatment groups generally showed an initial decrease followed by an increase and subsequent decline. All groups peaked on day 2, showing increases of 49.3%, 38.5%, 64.0%, and 87.1%, respectively, with the 2.5 mmol/L SNP group showing the most significant increase (**Figure 3c**).

### 4.4 PAL and HMGR activities in fresh roots of *P. lactiflora*

#### 4.4.1 PAL and HMGR activities in fresh roots of RRP-germplasm

**Figure 4:**
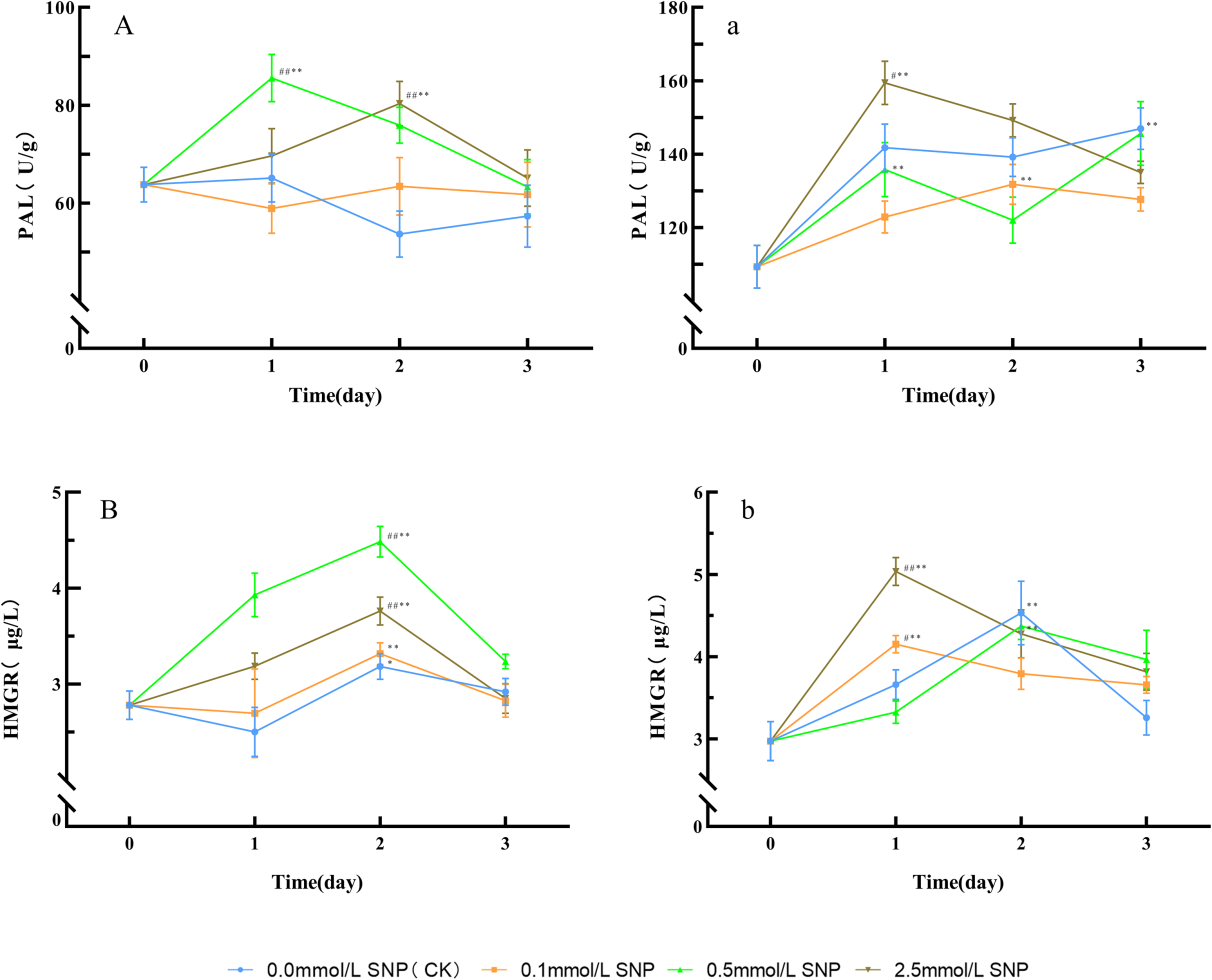
Changes in key enzyme activities in secondary metabolism in Paeoniae Radix Rubra **(A, B)** and Paeoniae Radix Alba **(a, b)** under different SNP concentrations. Data are presented as $\;\bar{x}$*± s* (*n* = 3, error bars: SD). Significance: */** vs day 0 (*P < 0.05, **P < 0.01); #/## vs 0.0 mmol/L SNP (#*P* < 0.05, ##*P* < 0.01). Groups: 0.0 mmol/L SNP (CK, blue), 0.1 mmol/L SNP (orange), 0.5 mmol/L SNP (green), 2.5 mmol/L SNP (brown). **(A, a)** PAL activities (U/g); **(B, b)** HMGR activities (μg/L).

Compared with day 0, the PAL activities in the water and 0.1 mmol/L SNP groups showed no significant changes, while the other two groups exhibited an initial increase followed by a decrease. The 0.5 mmol/L SNP group had the most pronounced enhancement in PAL activities, reaching its peak on day 1 with a 34.1% increase. The 2.5 mmol/L SNP group peaked on day 2 of treatment, showing a 26.0% elevation (**Figure 4A**). For HMGR activities, compared with day 0, the HMGR activities in the water and 0.1 mmol/L SNP groups showed minor fluctuations, while the other two groups exhibited an initial increase followed by a decrease. The 0.5 mmol/L SNP group had the most significant enhancement in HMGR activities. Both the 0.5 and 2.5 mmol/L SNP groups reached peaks on day 2, with increases of 61.2% and 35.3%, respectively (**Figure 4B**).

#### 4.4.2 PAL and HMGR activities in fresh roots of RAP-germplasm

Compared with day 0, the PAL activities in all groups showed an initial increase followed by a gradual decline. All groups reached their peaks on days 3, 2, 1, and 1, respectively, showing increases of 34.5%, 20.5%, 33.2%, and 45.8%, with the 2.5 mmol/L SNP group showing the most pronounced enhancement (**Figure 4a**). Regarding HMGR activities, compared with day 0, the HMGR activities in all groups initially increased and subsequently decreased. All groups peaked on days 2, 1, 2, and 1, respectively, with increases of 52.4%, 39.8%, 47.3%, and 69.6%, with the 2.5 mmol/L SNP group showing the most significant increase (**Figure 4b**). These results indicate that the application of an appropriate concentration of exogenous NO contributes to the increase in the activities of PAL and HMGR.

### 4.5 Level of secondary metabolites in fresh roots of *P. lactiflora*

#### 4.5.1 Level of secondary metabolites in fresh roots of RRP-germplasm

**Figure 5:**
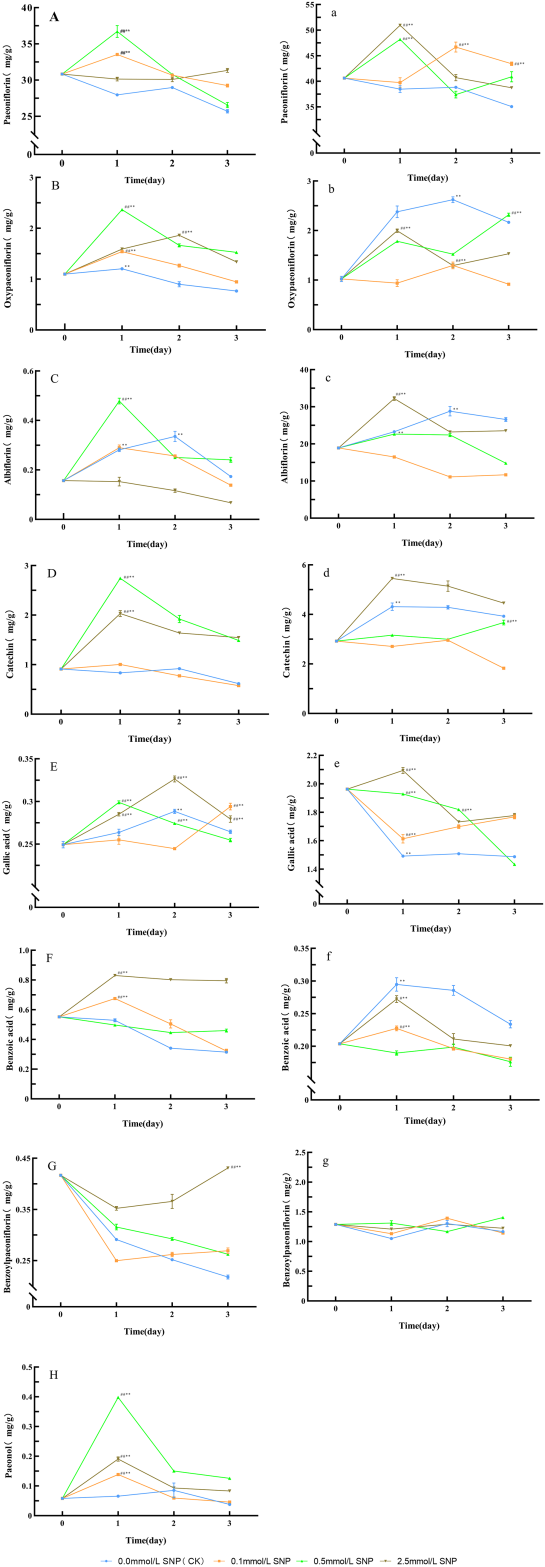
Changes in secondary metabolites levels in Paeoniae Radix Rubra **(A–H)** and Paeoniae Radix Alba**(a–g)** under different SNP concentrations. Data are presented as $\;\bar{x}$*± s* (*n* = 3, error bars: SD). Significance: */** vs day 0 (*P < 0.05, **P < 0.01); #/## vs 0.0 mmol/L SNP (#*P* < 0.05, ##*P* < 0.01). Groups: 0.0 mmol/L SNP (CK, blue), 0.1 mmol/L SNP (orange), 0.5 mmol/L SNP (green), 2.5 mmol/L SNP (brown). **(A, a)** Paeoniflorin content; **(B, b)** Oxypaeoniflorin content; **(C, c)** Albiflorin content; **(D, d)** Catechin content; **(E, e)** Gallic acid content; **(F, f)** Benzoic acid content; **(G, g)** Benzoylpaeoniflorin content; **(H)** Paeonal content (all units: mg/g).

Compared with day 0, the paeoniflorin in the water group showed a gradual decreasing trend, while the 2.5 mmol/L SNP group exhibited no significant changes, the remaining two groups exhibited an initial increase followed by a decrease, with the 0.5 mmol/L SNP group displaying the most pronounced elevation. Both the 0.1 and 0.5 mmol/L SNP groups reached their peaks on day 1, showing increases of 8.8% and 19.1%, respectively (**Figure 5A**). Regarding oxypaeoniflorin, compared with day 0, the oxypaeoniflorin in the water group showed a gradual decreasing trend, while all SNP-treated groups exhibited an initial increase followed by a decrease. The 0.1, 0.5, and 2.5 mmol/L SNP groups reached their peaks on days 1, 1, and 2 of treatment, respectively, showing increases of 40.2%, 115.4%, and 69.0%, the 0.5 mmol/L SNP group with the most significant elevation (**Figure 5B**). For albiflorin, compared with day 0, the albiflorin in the 2.5 mmol/L SNP group showed a gradual decreasing trend, while the other three groups exhibited an initial increase followed by a decrease. The 0.0, 0.1, and 0.5 mmol/L SNP groups reached their peaks on days 2, 1, and 1 of treatment, respectively, with increases of 114.3%, 85.0%, and 205.4%, the 0.5 mmol/L SNP group with the most significant increase (**Figure 5C**). As for catechin, compared with day 0, the catechin in the water and 0.1 mmol/L SNP groups showed no significant changes, while the other two groups exhibited an initial increase followed by a decreasing trend. Both the 0.5 and 2.5 mmol/L SNP groups reached their peaks on day 1, showing increases of 201.0% and 122.7%, respectively, the 0.5 mmol/L SNP group with the most pronounced elevation (**Figure 5D**). In terms of gallic acid, compared with day 0, the gallic acid in the water and 0.1 mmol/L SNP groups showed no significant changes, while the other two groups exhibited an initial increase followed by a decreasing trend. The 0.5 and 2.5 mmol/L SNP groups reached their peaks on days 1 and 2 of treatment, respectively, showing increases of 19.9% and 30.9%, respectively, the 2.5 mmol/L SNP group with the most pronounced increase (**Figure 5E**). Benzoic acid showed a different pattern; compared with day 0, the benzoic acid in the water group showed a gradual decreasing trend. The 0.5 mmol/L SNP group exhibited no significant changes, while the other two groups exhibited an initial increase followed by a decrease. Both the 0.1 and 2.5 mmol/L SNP groups reached their peaks on day 1, showing increases of 22.3% and 50.4%, respectively, the 2.5 mmol/L SNP group displaying the most pronounced increase (**Figure 5F**). However, benzoylpaeoniflorin differed from the above substances in its trend; compared with day 0, the benzoylpaeoniflorin in the 2.5 mmol/L SNP group showed an initial decrease followed by an upward trend, returning to the baseline level on day 3 of treatment, while the other three groups exhibited a continuous downward trend throughout the experimental period (**Figure 5G**). Finally, for paeonol, compared with day 0, the paeonol in the water group showed a gradual decreasing trend, while all SNP-treated groups exhibited an initial increase followed by a decrease. The 0.1, 0.5, and 2.5 mmol/L SNP groups all reached peaks on day 1 of treatment, showing increases of 138.3%, 585.2%, and 228.1%, respectively, the 0.5 mmol/L SNP group with the most significant elevation (**Figure 5H**). These results indicate that spraying 0.5 mmol/L SNP solution has a relatively good effect on increasing the secondary metabolites in the fresh roots of RRP-germplasm.

#### 4.5.2 Level of secondary metabolites in fresh roots of RAP-germplasm

Compared with day 0, the paeoniflorin in the water group showed a gradual decreasing trend, while the other three groups exhibited an initial increase followed by a decrease. The 0.1, 0.5, and 2.5 mmol/L SNP groups reached their peaks on days 2, 1, and 1, respectively, with increases of 15.0%, 18.6%, and 25.4%, respectively, the 2.5 mmol/L SNP group with the most significant elevation (**Figure 5a**). Regarding oxypaeoniflorin, compared with day 0, the 0.1 mmol/L SNP group showed no significant change in oxypaeoniflorin contents, while the 0.5 mmol/L SNP group exhibited a gradual increasing trend, the remaining two groups exhibiting an initial increase followed by a decrease. The 0, 0.5, and 2.5 mmol/L groups reached their peaks on days 2, 3, and 1 of treatment, respectively, showing increases of 156.4%, 126.7%, and 95.1%, respectively, the water group showing the most pronounced elevation (**Figure 5b**). For albiflorin, compared with day 0, the albiflorin in the 0.1 mmol/L SNP group showed a gradual decreasing trend, while the other three groups exhibited an initial increase followed by a decrease. The 0.0, 0.5, and 2.5 mmol/L SNP groups reached their peaks on days 2, 1, and 1 of treatment, respectively, with increases of 52.4%, 19.9%, and 70.4%, respectively, the 2.5 mmol/L SNP group with the most significant increase (**Figure 5c**). As for catechin, compared with day 0, the catechin in the 0.1 and 0.5 mmol/L SNP groups showed no significant change trend, while the remaining two groups exhibited an initial increase followed by a decrease. Both the 0.0 and 2.5 mmol/L SNP groups reached their peaks on day 1, showing increases of 47.4% and 86.5%, respectively, the 2.5 mmol/L SNP group with the most pronounced elevation (**Figure 5d**). In terms of gallic acid, compared with day 0, only the 2.5 mmol/L SNP group exhibited an initial increase followed by a decrease in gallic acid level, reaching its peak on day 1 of treatment with a 6.7% increase compared to day 0. The other three groups generally showed a downward trend (**Figure 5e**). For benzoic acid, compared with day 0, the 0.1 mmol/L SNP group showed no significant change in benzoic acid contents, while the other three groups exhibited an initial increase followed by a decreasing trend. The peaks for the 0, 0.5, and 2.5 mmol/L SNP groups were all observed on day 1 of treatment, showing increases of 44.7%, 11.6%, and 33.6%, respectively, the water group with the most pronounced elevation (**Figure 5f**). Finally, for benzoylpaeoniflorin, compared with day 0, the level of benzoylpaeoniflorin in all groups showed no significant change trend, with relatively minor fluctuations overall (**Figure 5g**). These results indicate that spraying 2.5 mmol/L SNP solution has a relatively good effect on increasing the secondary metabolites in the fresh roots of RAP-germplasm.

### 4.6 1,3-DPG level in fresh roots of *P. lactiflora*

#### 4.6.1 1,3-DPG level in fresh roots of RRP-germplasm

**Figure 6:**
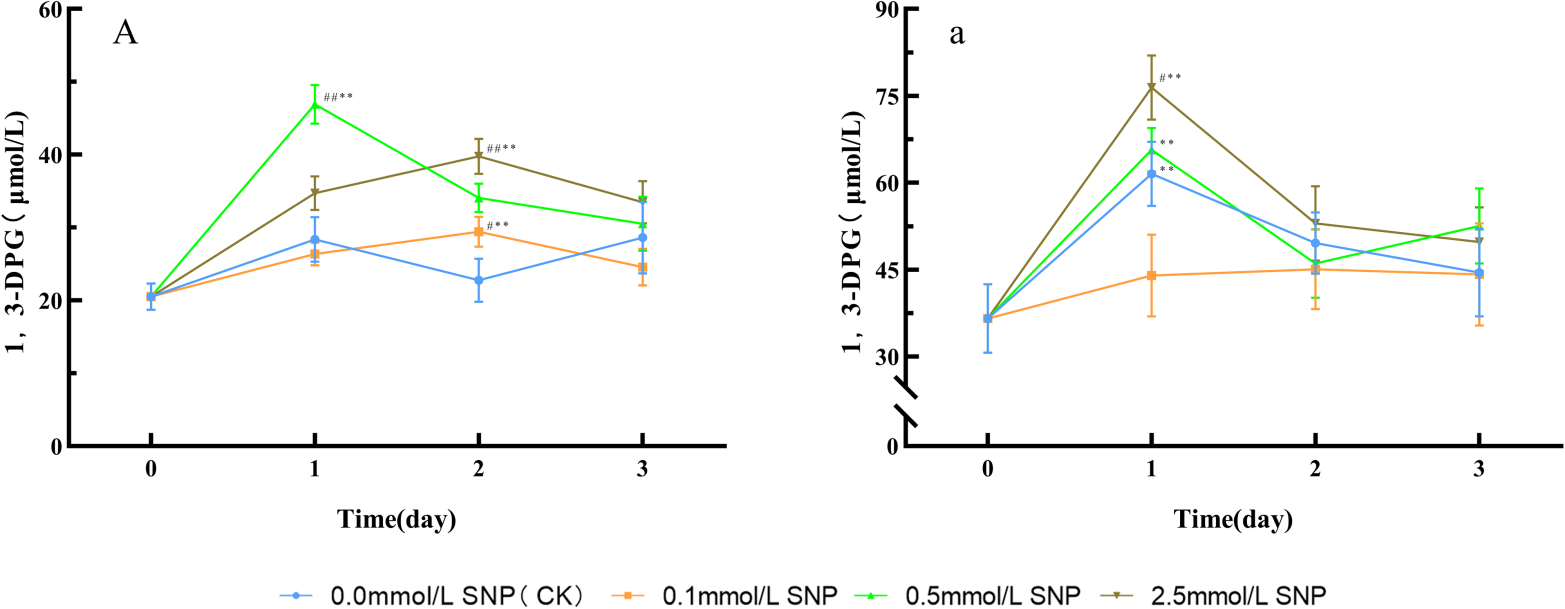
Changes in 1,3-DPG levels in Paeoniae Radix Rubra **(A)** and Paeoniae Radix Alba **(a)** under different SNP concentrations. Data are presented as $\;\bar{x}$*± s* (*n* = 3, error bars: SD). Significance: */** vs day 0 (*P < 0.05, **P < 0.01); #/## vs 0.0 mmol/L SNP (#*P* < 0.05, ##*P* < 0.01). Groups: 0.0 mmol/L SNP (CK, blue), 0.1 mmol/L SNP (orange), 0.5 mmol/L SNP (green), 2.5 mmol/L SNP (brown). A/a: 1,3-DPG contenst (μmol/L).

Compared with day 0, the 1,3-DPG contents in the water group showed no significant change trend, while all other groups exhibited an initial increase followed by a decrease. The 0.5 mmol/L SNP group demonstrated the most pronounced increase, reaching its peak on day 1 with a 128.8% elevation. In contrast, both the 0.1 and 2.5 mmol/L SNP groups peaked on day 2, showing increases of 43.4% and 93.7%, respectively (**Figure 6A**).

#### 4.6.2 1,3-DPG level in fresh roots of RAP-germplasm

Compared with day 0, the 0.1 mmol/L SNP group showed no significant changes in 1,3-DPG contents, while the other groups exhibited a trend of initial increase followed by a decrease. The 0.0, 0.5, and 2.5 mmol/L SNP groups reached peaks on day 1 of treatment, with increases of 68.3%, 79.6%, and 109.1%, respectively, the 2.5 mmol/L SNP group with the most pronounced elevation (**Figure 6a**).

### 4.7 Effect of SNP and ROS scavengers on ROS and MDA

When RRP-germplasm fresh roots were treated with 0.5 mmol/L SNP alone, the levels of O_2_·^-^, H_2_O_2_, and MDA were significantly higher than those in the control group, indicating that SNP successfully induced ROS outburst in fresh roots. However, after the subsequent application of 0.1 mmol/L *α*-Toc and 1.0 mmol/L NAC following SNP treatment, the levels of O_2_·^-^, H_2_O_2_, and MDA in fresh roots were significantly decreased compared with those in the 0.5 mmol/L SNP alone group, closer to the levels in the control group. These results demonstrated that *α*-Toc and NAC could effectively scavenge the ROS induced by SNP in RRP-germplasm fresh roots.

### 4.8 Dependence of SNP-induced secondary metabolism on ROS

When RRP-germplasm fresh roots were treated with 0.5 mmol/L SNP alone, the levels of paeoniflorin, albiflorin, oxypaeoniflorin, gallic acid, catechin, and paeonol were significantly higher than those in the control group, indicating that SNP could effectively induce secondary metabolism in fresh roots. When 0.1 mmol/L *α*-Toc and 1.0 mmol/L NAC were subsequently applied after SNP treatment, the levels of these secondary metabolites were significantly lower than those in the 0.5 mmol/L SNP alone group, closer to the levels in the control group, indicating that the inductive effect of SNP on the secondary metabolism of RRP-germplasm depended on ROS accumulation.

## 5 Discussion

When plants are subjected to environmental stress, their cells maintain ROS homeostasis through multidimensional mechanisms, including enzymatic activity regulation, enhanced secondary metabolism, and osmotic balance adjustment. As scavengers of ROS and cytoprotective agents, secondary metabolites serve as core defensive compounds in plants, and become the active pharmaceutical ingredients of medicinal herbs. The formation of the quality of herbal medicines is essentially the process of biosynthesis of secondary metabolites triggered by environmental stress signals, more accurately, it is a process of plant adaptation to ecological stress. Therefore, the content of pharmacologically active ingredients can be improved by constructing the physiological state of plants under adverse conditions.

### 5.1 Effect of SNP on ROS and MDA levels

The impact of different stresses on ROS in plants varies depending on the stress type and plant species, but they all lead to a significant increase in ROS levels. For instance, under salt stress, the H_2_O_2_ level in *Cucumis sativus* L. roots increases by approximately 30.0% after 1 hour of treatment (Kabała et al., 2022). Low-temperature treatment increases H_2_O_2_ levels in strawberry leaves (*Fragaria × ananassa* Duch.) by nearly 2-fold (Ya et al., 2011). High-temperature stress causes the O_2_·^-^ levels in wheat (Triticum aestivum L.) to rise by 50.0% (Pradhan and Prasad, 2015). NO can impair complexes III and IV in the mitochondrial electron transport chain, thereby exacerbating electron leakage and promoting the generation of O_2_·^-^. In addition, NO can directly activate the plasma membrane nicotinamide adenine dinucleotide phosphate hydrogen (NADPH) oxidase or via the calcium-protein kinase–mediated signaling cascade, thereby inducing the production of ROS (Delledonne et al., 1998b; Corpas et al., 2011; Yun et al., 2011). High concentrations of ROS can initiate free radical chain reactions in membrane lipid peroxidation, damaging the ultrastructure of plant cells and inducing membrane denaturation, which leads to reduced selective permeability, disruption of cellular compartmentalization, and an imbalance in material exchange between the cell and its environment, ultimately leading to cellular dysfunction (Pratt et al., 2011). As a key toxic byproduct of membrane lipid peroxidation, MDA can further induce protein cross-linking and DNA damage, so it has become a critical indicator for assessing oxidative cellular damage and stress resistance (Peng et al., 2022). **Figure 1** shows that SNP rapidly increased O_2_·^-^ levels in *P. lactiflora* fresh roots, followed by a swift rise in H_2_O_2_ level; subsequently, MDA contents in all groups showed significant elevation (**Figure 2**), indicating that exogenous NO can induce a rapid short-term increase in ROS, and cause damage to plant cells. SNP can replicate the physiological state of plants under stress conditions.

### 5.2 Effects of SNP on antioxidant enzyme activities

The ROS scavenging system in plants consists of enzymatic and non-enzymatic components, and they dynamically complement each other to form a precisely regulated antioxidant network (Ahmad et al., 2010; Wang et al., 2024). O_2_·^-^ is the earliest ROS generated and readily induces the Fenton reaction to produce ·OH, a kind of ROS with the highest toxicity, and causes severe cellular damage (Phua et al., 2021). SOD, as the only enzyme capable of specifically catalyzing O_2_·^-^, forms the first line of antioxidant defense (Case, 2017). Taking Cu-Zn SOD as an example, when O_2_·^-^ levels rise, Cu²^+^ is reduced to Cu^+^, catalyzing the disproportionation of two O_2_·^-^ into H_2_O_2_ and O_2_. This substrate-inducible characteristic ensures that SOD activity exhibits a positive correlation with O_2_·^-^ within physiological ranges. Since its catalytic efficiency directly determines the subsequent cascade reactions of CAT and POD, SOD plays a central role in ROS metabolism (Sheng et al., 2014). H_2_O_2_ can readily penetrate biological membranes and react with cytoplasmic components, oxidizing thiol groups and metal cofactors, thereby inactivating proteins and enzymes while impairing NADPH oxidase. This process exacerbates the collapse of the antioxidant system (Kunkemoeller and Kyriakides, 2017). CAT, a tetrameric enzyme containing iron porphyrin as its prosthetic group (Baker et al., 2023), decomposes H_2_O_2_ into H_2_O and O_2_ via a disproportionation reaction. It requires the successive collisions of two H_2_O_2_ at the active site to initiate the reaction, making CAT the most specialized and direct enzyme for H_2_O_2_ clearance (Yang and Poovaiah, 2002). Unlike CAT, POD is a heme-rich oxidoreductase. Its heme group can undergo a cyclic transition between the oxidized (Fe³^+^) and reduced (Fe²^+^) states, enabling it to withstand intense oxidative stress from ROS (Bauer and Bauer, 2002). Furthermore, the hydrophobic cavity within its tertiary structure shields the active site from radical attacks (Gray and Winkler, 2021). Therefore, even when ROS concentrations increase, POD still maintains excellent stability (Amin et al., 2024). Under stress conditions, certain secondary metabolites—such as phenolic compounds (catechin, paeonol, and baicalein)—can serve as substrates to supply electrons and facilitate the continuous reduction of H_2_O_2_ by POD (Niu and Liao, 2016).

SNP increased ROS levels, and modulated protein disulfide bonds, and enhanced antioxidant enzyme activity (**Figures 1**, **3**). In both types of fresh *P. lactiflora* roots, SOD activities significantly increased within 0~1 day after SNP exposure (**Figure 3**), promoting O_2_·^-^ reduction in all groups by days 2~3 (**Figure 1**). CAT and POD showed synergistic and complementary effects in the clearance of H_2_O_2_. When the activity of one enzyme decreased, the activity of another enzyme increased correspondingly. In the fresh roots of the RRP-germplasm, CAT and POD activities peaked at days 1 and 2, respectively, after SNP treatment, whereas in the RAP-germplasm, CAT activity peaked during days 1~2, and POD activity reached a peak on day 2. The synergistic action of both enzymes effectively reduced H_2_O_2_ levels (**Figure 1**). Notably, POD maintained persistently high activity during the late stress phase (days 2~3) in all treated groups, indicating that POD plays a critical role in H_2_O_2_ clearance (**Figure 3**), leading to a reduction in ROS levels in the fresh roots of the two *P. lactiflora* germplasms. However, as the stress intensity increased or the duration of stress was extended, the activities of various antioxidant enzymes declined rapidly within 2~3 days of treatment (**Figure 3**). This phenomenon may be attributed to the high ROS, which oxidize amino acid residues in enzyme proteins, such as cysteine, methionine, and tyrosine, disrupt the homeostasis of disulfide bonds (-S-S-) or induce peptide chain cross-linking, and further cause the dissociation of metal cofactors—including Cu²^+^ in Cu/Zn-SOD and Fe²^+^ in CAT—from the active center. These changes collectively destroy the spatial conformation of antioxidant enzymes such as SOD, CAT, and POD, thereby inactivating their catalytic functions (Karamat et al., 2024). **Figure 2** shows that MDA contents remained consistently elevated, suggesting that the decline in antioxidant enzyme activity may indeed be caused by ROS. These observations collectively suggest that antioxidant enzymes may not be the primary means of ROS clearance under severe stress conditions, implying potential contributions from secondary metabolites.

### 5.3 SNP modulates secondary metabolites in *P. lactiflora*

**Figure 7:**
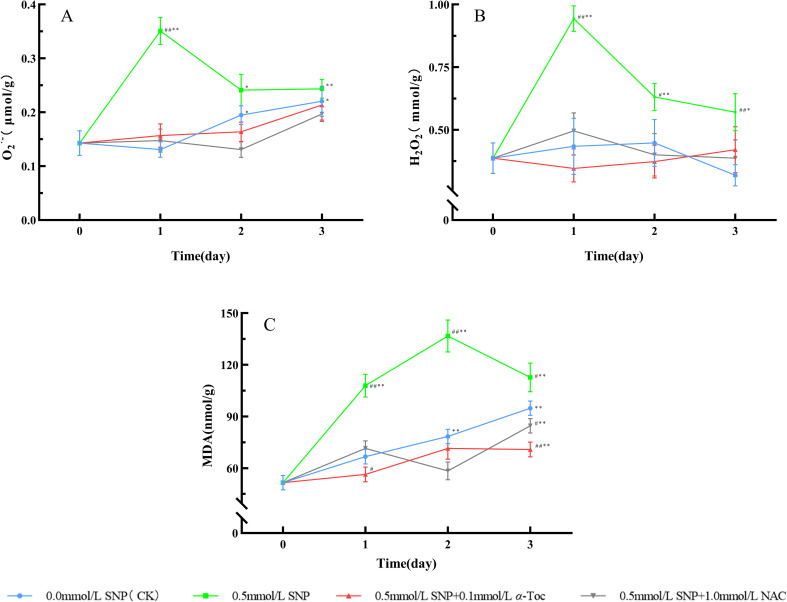
ROS Levels and MDA Changes in Paeoniae Radix Rubra **(A–C)**. Data are presented as $\;\bar{x}$*± s* (*n* = 3, error bars: SD). Significance: */** vs day 0 (*P < 0.05, **P < 0.01); #/## vs 0.0 mmol/L SNP (#*P* < 0.05, ##*P* < 0.01).Groups: 0.0 mmol/L SNP (CK, blue), 0.5 mmol/L SNP (green), 0.5 mmol/L SNP + 0.1 mmol/L *α*-Toc(red), 0.5 mmol/L SNP + 1.0 mmol/L NAC (gray). **(A)** O_2_·^-^ contents (μmol/g); **(B)** H_2_O_2_ contents (mmol/g); **(C)** MDA contents (nmol/g).

**Figure 8:**
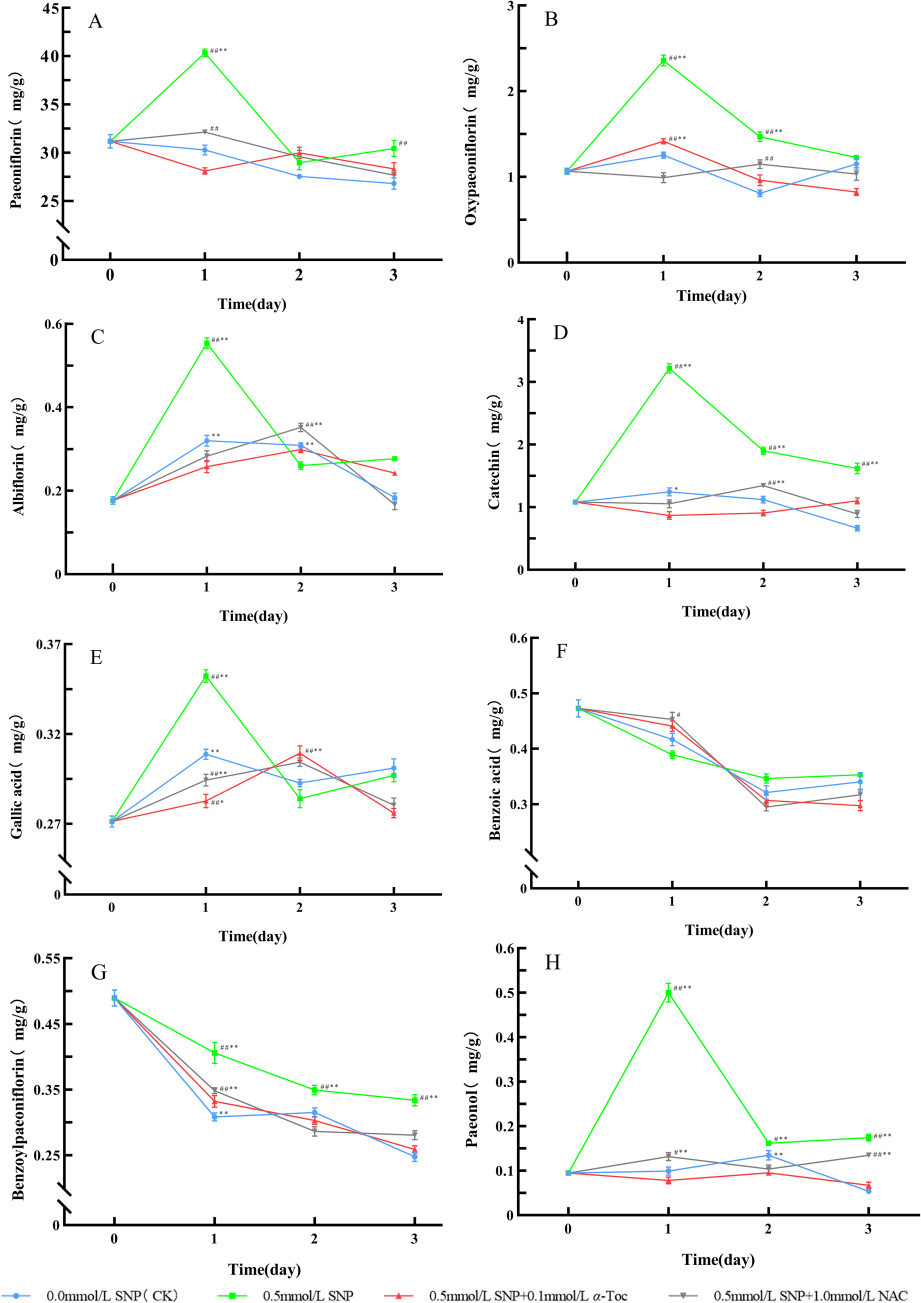
Changes in Secondary Metabolites Levels in Paeoniae Radix Rubra **(A–H)**. Data are presented as $\;\bar{x}$*± s* (*n* = 3, error bars: SD).Significance: */** vs day 0 (*P < 0.05, **P < 0.01); #/## vs 0.0 mmol/L SNP (#*P* < 0.05, ##*P* < 0.01).Groups: 0.0 mmol/L SNP (CK, blue), 0.5 mmol/L SNP (green), 0.5 mmol/L SNP + 0.1 mmol/L *α*-Toc(red), 0.5 mmol/L SNP + 1.0 mmol/L NAC (gray) **(A)** Paeoniflorin content; **(B)** Oxypaeoniflorin content; **(C)** Albiflorin content; **(D)** Catechin content; **(E)** Gallic acid content; **(F)** Benzoic acid content; **(G)** Benzoylpaeoniflorin content; **(H)** Paeonol content (all units: mg/g).

Unlike animals, plants cannot escape adverse conditions, which inevitably leads to the overproduction of ROS (Ali et al., 2023). In response, plants have evolved a unique strategy to cope with stress, involving activating gene expression, regulating the activity of antioxidant enzymes, and reconstructing primary and secondary metabolic pathways. When the ROS generated exceed the scavenging capacity of the antioxidant enzyme system, plants enhance their secondary metabolites to protect plant tissues from oxidative stress (Miller et al., 2010). The main secondary metabolites of *P. lactiflora* include monoterpenes and phenolic compounds, such as paeoniflorin, albiflorin, oxypaeoniflorin, gallic acid, catechin, paeonol, and benzoylpaeoniflorin. Among them, catechin, gallic acid, and paeonol belong to different subclasses of phenolic compounds, all containing phenolic hydroxyl groups, with the ability to scavenge H_2_O_2_, inhibit NADPH oxidase (Ma et al., 2024b), chelate transition metal ions such as Fe²^+^ and Cu²^+^, block the Fenton reaction, and suppress the generation of ROS (Kubo et al., 2010), thereby reducing the oxidative damage caused by ROS to DNA and proteins, and protecting the enzyme system from damage (Fraga et al., 2019). The monoterpene compound paeoniflorin possesses electrophilicity due to its conjugated benzene ring system and *β*-D-glucopyranosyl group, and can directly capture and neutralize free radicals (Nakayama and Uno, 2024; Zhao et al., 2024). As an isomer of paeoniflorin, albiflorin contains an ester glycoside group that confers strong electron-donating capacity, and it can not only directly eliminate ROS, but also maintain the function of mitochondrial membrane potential and electron transport chain, so as to reduce the production of ROS caused by electron leakage (Suh et al., 2013). Because the synthesis of secondary metabolites consumes substantial amounts of NADPH and ATP, the continuous production of secondary metabolites in plants under suitable conditions will significantly inhibit their growth and development. Only under stress can the plants’ defense response be activated and energy resources reallocated for the production of defensive compounds (Pant et al., 2021), resulting in secondary metabolites being synthesized in large quantities (Ozyigit et al., 2023). When plants are subjected to environmental stresses such as pathogen infection, ultraviolet radiation, or mechanical damage, ROS activate the phenylpropanoid pathway and enhance the activity of PAL (Moghaddam et al., 2019; Wang et al., 2019), a key rate-limiting enzyme for flavonoid synthesis (MacDonald and D’Cunha, 2007). For instance, under salt stress, the activation of PAL in *Brassica oleracea* L. stimulates the production of flavonoid compounds such as rutin and quercetin (Yang et al., 2018). HMGR is a rate-limiting enzyme involved in the synthesis of the monoterpenoid skeleton in the mevalonate pathway (Zheng et al., 2022). It can also be regulated by ROS and enhance the biosynthesis of paeoniflorin, albiflorin, benzoylpaeoniflorin, and oxypaeoniflorin in *P. lactiflora* (Xu et al., 2023). Under drought stress, the tanshinone and cryptotanshinone in *Salvia miltiorrhiza* Bunge increase remarkably (Yang et al., 2018); the atractylodin, atractylon, and atractylenolide II are substantially elevated in *Atractylodes chinensis* (DC.) Koidz (Zhao et al., 2023). The secondary metabolites of medicinal plants are usually medicinal components. **Figures 7**, **8** show that the inductive effect of SNP on the secondary metabolism of RRP-germplasm depended on the ROS accumulation it induced, and this inductive effect was significantly attenuated when antioxidants scavenged ROS, which confirmed that increased ROS under stress conditions was the fundamental cause of enhanced secondary metabolism.

**Figure 4** shows that the SNP enhanced the activities of PAL and HMGR, resulting in increased flavonoids and monoterpenoids in all treatment groups (**Figure 5**). During SNP treatment, the intracellular ROS levels exhibited a trend consistent with PAL and HMGR activities, both of which increased first and then decreased. Catechin, paeoniflorin, albiflorin, and oxypaeoniflorin in the 0.5 mmol/L SNP group of RRP-germplasm and the 2.5 mmol/L SNP group of RAP-germplasm increased by 201.0%, 19.1%, 205.4%, 115.4% and 86.5%, 25.4%, 70.4%, 95.1%, respectively, on the first day of treatment (**Figure 5**), which indicates the difference in the responses of different germplasms of the same species to SNP. In RRP-germplasm, the 2.5 mmol/L SNP group showed poor enhancement effects, maybe due to the higher ROS damaging PAL and HMGR (**Figure 4**), while the 0.0 and 0.1 mmol/L SNP groups had lower ROS, insufficient to fully activate the self-defense system (**Figures 3**, **4**), and limited secondary metabolite synthesis. In RAP-germplasm, however, the 2.5 mmol/L SNP group exhibited the highest activity of PAL and HMGR, with the overall secondary metabolites significantly exceeding those of the 0.1 and 0.5 mmol/L SNP groups, suggesting that RAP-germplasm possesses stronger stress tolerance compared to RRP-germplasm. **Figure 5** shows that excess H_2_O as a form of adversity stress can also enhance ROS accumulation in RAP-germplasm and increase some secondary metabolites, but has a very limited effect on the improvement of major active ingredients such as paeoniflorin and albiflorin, the effect of which is much lower than that of the 2.5 mmol/L SNP group (*P* < 0.01).During the treatment of *P. lactiflora* fresh roots, with the enhancement of the synthesis of related antioxidants, especially secondary metabolites, ROS decreased rapidly (**Figures 1**, **3**–**5**), indicating that secondary metabolites play an important role in adapting to severe stress.

Phenylalanine and isopentenyl pyrophosphate are both intermediates linking primary and secondary metabolism, and the enhanced activities of PAL and HMGR (**Figure 4**) will lead to more primary metabolites being used for the biosynthesis of secondary metabolites. 1,3-DPG is a metabolic intermediate in glycolysis, and the increase in its content reflects an enhancement of glycolytic flux (Muñoz-Bertomeu et al., 2010). This study used isolated roots of *P. lactiflora*, with no material originating from photosynthesis; thus, the increased 1,3-DPG must have derived from the breakdown of sugars. The increased paeoniflorin, albiflorin, catechin, and other components were synthesized from non-pharmaceutical carbohydrates; thus, the quality of the drug improved significantly.

### 5.4 Effects of SNP on quality and specific compound accumulation in *P. lactiflora*

A plant species with a wide distribution can form multiple ecotypes due to diverse ecological conditions, generating different germplasms which exhibit significantly varied responses to specific environmental stresses, including drought, saline-alkaline stress, high-temperature stress, and biotic stresses. After long-term natural selection and artificial selection, the same plant species has formed unique physiological and biochemical adaptation mechanisms, and its secondary metabolism has changed (Jiang et al., 2025). In this study, the RRP-germplasm treated with 0.5 mmol/L SNP increased paeoniflorin, catechin, and paeonol contents by 19.1%, 201.0%, and 585.2%, respectively; the RAP-germplasm treated with 2.5 mmol/L SNP markedly increased albiflorin and oxypaeoniflorin contents by 70.4% and 95.1%, respectively. This study showed that SNP had different effects on different germplasms of the same species, but both germplasms could significantly improve the quality of medicinal materials.

## 6 Conclusion

The active components of medicinal plants are generally secondary metabolites of plants, and the quality formation mechanism of medicinal plants is essentially the adaptation mechanism of plants to ecological stress. This study successfully simulated the physiological state of plants under ecological stress conditions by using exogenous NO. In fresh roots of *P. lactiflora*, the ROS content increased, and the antioxidant enzyme activities as well as the content of secondary metabolites increased significantly; these two factors acted synergistically to scavenge excess ROS and reduce the damage caused by ROS to the plant. However, with the increase in stress intensity, antioxidant enzyme activities decreased, while the activities of key secondary metabolism enzymes increased, which promoted the biosynthesis of specific medicinal components, thereby improving the quality of cultivated *P. lactiflora*. Specifically, in the fresh roots of RRP-germplasm treated with 0.5 mmol/L SNP, the secondary metabolites paeoniflorin, albiflorin, oxypaeoniflorin, gallic acid, catechin, and paeonol were elevated by 19.1%, 205.4%, 115.4%, 19.9%, 201.0%, and 585.2%, respectively; in the fresh roots of RAP-germplasm treated with 2.5 mmol/L SNP, the major secondary metabolites paeoniflorin, albiflorin, oxypaeoniflorin, gallic acid, catechin, and benzoic acid showed increases of 25.4%, 70.4%, 95.1%, 6.7%, 86.5%, and 33.6%, respectively. Notably, the magnitude of the increase in active ingredient content observed in this study has rarely been reported in previous research. Compared with field plant treatments that are difficult to precisely control, isolated fresh root tissue treatment has significant advantages such as active metabolism, sensitive response, controllable conditions, and prevention of irreversible damage to the plant. This *in vitro* targeted induction model exhibits greater superiority and feasibility in increasing the content of target components, providing an innovative technical approach for the standardized and high-quality production of *P. lactiflora*.

## Data Availability

The original contributions presented in the study are included in the article/**Supplementary Material**. Further inquiries can be directed to the corresponding author/s.
