# Supplementary material for: Biological mechanisms of sodium nitroprusside in enhancing quality of Radix Paeoniae Rubra and Radix Paeoniae Alba
Source: Front Plant Sci. 2025 Nov 12;16:1660058. doi: 10.3389/fpls.2025.1660058 (PMC12646931; doi:10.3389/fpls.2025.1660058)
Supplement: Supplementary file 1 [file DataSheet1.docx]

Suppl. Fig. 1 HPLC chromatograms of different samples.

The separation was performed on a Diamonsil C₁₈ column (250 mm × 4.6 mm, 5 μm) using a mobile phase consisting of acetonitrile (A) and a pH 2.7 phosphoric acid aqueous solution (B) under gradient elution as follows: 0–20 min, 5% A to 15% A; 20–40 min, 15% A to 20% A; 40–50 min, 20% A; 50–80 min, 20% A to 40% A; 80–90 min, 40% A to 5% A; 90–100 min, 5% A. The column temperature was maintained at 25 °C, with an injection volume of 10 μL and a flow rate of 1 mL/min. Detection was carried out at a wavelength of 230 nm. **A** represents the chromatogram of mixed reference substances; **B** represents the chromatogram of *Paeoniae Radix Rubra* samples; **C** represents the chromatogram of *Paeoniae Radix Alba* samples. Peaks correspond to the following compounds: 1. Gallic acid; 2. Oxypaeoniflorin; 3. Catechin; 4. Albiflorin; 5. Paeoniflorin; 6. Benzoic acid; 7. Benzoylpaeoniflorin; 8. Paeonal.

Table 1. The linear relationship of 8 components.


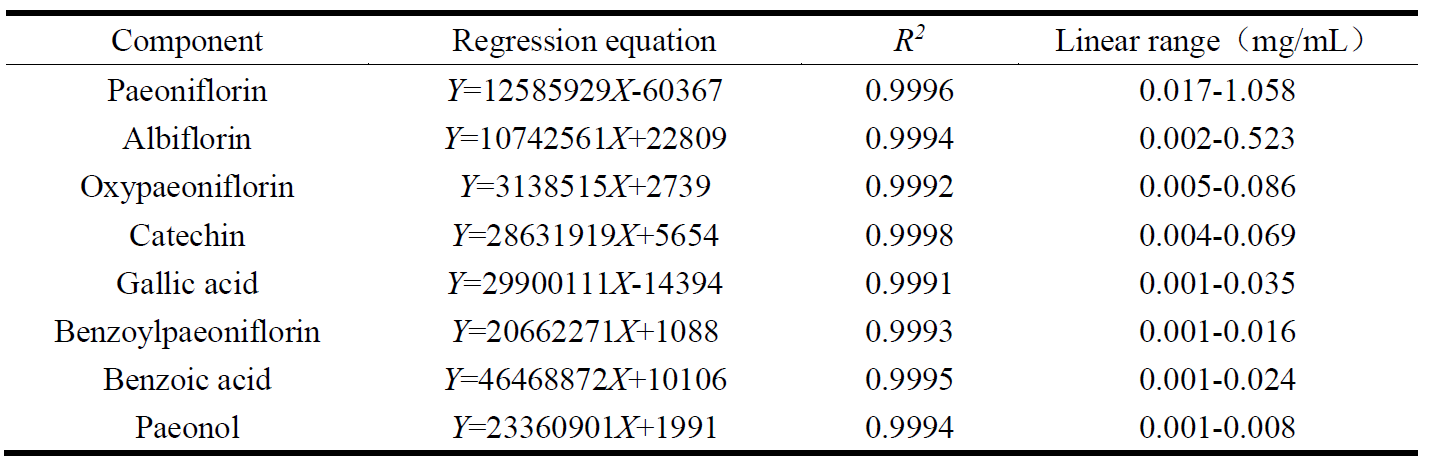


Under the aforementioned chromatographic conditions, the solutions were injected, and the peak areas of paeoniflorin, albiflorin, oxypaeoniflorin, gallic acid, catechin, paeonol, benzoic acid, and benzoylpaeoniflorin were recorded. Regression analysis was performed using the mass concentration as the abscissa (*X*) and the peak area as the ordinate (*Y*). The regression equations, coefficient of determination (*R^2^*), and linear ranges for the eight components were calculated, demonstrating good linearity within the specified ranges.
